# Mathematical simulation of tumour angiogenesis: angiopoietin balance is a key factor in vessel growth and regression

**DOI:** 10.1038/s41598-020-79824-8

**Published:** 2021-01-11

**Authors:** Hayato Yanagisawa, Masahiro Sugimoto, Tomoyuki Miyashita

**Affiliations:** 1 Faculty of Science and Engineering, Waseda University, 3-4-1 Okubo, Shinjuku-ku, Tokyo, 169-8555 Japan; 2 Research and Development Centre for Minimally Invasive Therapies, Medical Research Institute, Tokyo Medical University, 6-1-1, Shinjuku, Tokyo, 160-0022 Japan; 3 Institute for Advanced Biosciences, Keio University, Yamagata, 997-0811 Japan

**Keywords:** Computational models, Tumour angiogenesis, Computational biology and bioinformatics

## Abstract

Excessive tumour growth results in a hypoxic environment around cancer cells, thus inducing tumour angiogenesis, which refers to the generation of new blood vessels from pre-existing vessels. This mechanism is biologically and physically complex, with various mathematical simulation models proposing to reproduce its formation. However, although temporary vessel regression is clinically known, few models succeed in reproducing this phenomenon. Here, we developed a three-dimensional simulation model encompassing both angiogenesis and tumour growth, specifically including angiopoietin. Angiopoietin regulates both adhesion and migration between vascular endothelial cells and wall cells, thus inhibiting the cell-to-cell adhesion required for angiogenesis initiation. Simulation results showed a regression, i.e. transient decrease, in the overall length of new vessels during vascular network formation. Using our model, we also evaluated the efficacy of administering the drug bevacizumab. The results highlighted differences in treatment efficacy: (1) earlier administration showed higher efficacy in inhibiting tumour growth, and (2) efficacy depended on the treatment interval even with the administration of the same dose. After thorough validation in the future, these results will contribute to the design of angiogenesis treatment protocols.

## Introduction

Cancer is a leading cause of death globally, with more than 9 million deaths per year; a number which is still increasing^1,2^. In the 1970s, Folkman discovered tumour-induced angiogenesis, i.e. the sprouting of new blood vessels from existing blood vessels, on which the growth and necrosis of solid tumours depends^3^. Both excessive tumour cell proliferation and lack of blood vessels induce hypoxia in the tumour microenvironment^4^. Tumour and the cells surrounding it secret tumour angiogenesis factors (TAFs), which stimulate existing blood vessels to induce angiogenesis^5^. Through angiogenesis, tumours gain a new vascular network, enabling them to receive nutrition, and metastasize and infiltrate other tissues^6^. The tumour microenvironment, such as TAF distribution and extracellular matrix (ECM), is central to the formation of these vascular networks^7^. As the complicated mechanisms underpinning tumour-induced angiogenesis are affected by both physical and chemical reactions, their specific details have not yet been fully elucidated.

Bevacizumab (product name: Avastin) has been approved by the U.S. Food and Drug Administration for the treatment of metastatic colon cancer, certain lung cancers, renal cancers, ovarian cancers, and glioblastoma multiforme of the brain. Bevacizumab is a monoclonal antibody which targets vascular endothelial growth factor (VEGF), which is considered to be the most important tissue factor among TAFs that are responsible for blood vessel formation^8^. It specifically binds to VEGF, inhibiting its activity and consequently suppressing angiogenesis^9^. Bevacizumab is usually administered in combination with other chemotherapeutic agents^10^. For example, in a clinical trial of patients with non-small cell lung cancer (NSCLC), overall survival (OS) increased significantly from 10.3 months with carboplatin-paclitaxel alone, to 12.3 months when used in combination with bevacizumab^11^. Thus, understanding of the biological relationship among tumours and their environment including VEGF is important for enhancing of therapeutic effects of bevacizumab.

Over the past few decades, mathematical simulation models of tumour angiogenesis have been extensively developed to elucidate its mechanisms or to predict cancer progression and the therapeutic effects of drugs^12,13^. Proposed models were classified into three types. The first only reproduced the phenomena of angiogenesis^14–18^. Anderson et al. developed a model that integrated both continuous and discontinuous models to describe the formation of capillary sprout networks in response to chemical stimuli from solid tumours^19^. The second described the tumour growth phenomena^20–23^. This type of model reproduced tumour growth and invasion when nourished by surrounding angiogenesis. For example, the diffuse interface continuum model was developed to describe the dependency of the tumour shape on cell-to-cell adhesive strength^24^. The third integrated tumour growth and angiogenesis^25–30^. Recently, various three-dimensional models have been reported due to improvements in computing performance^31–33^. Both tumour invasion and angiogenesis were combined in a three-dimensional model to find that tumours decompose surrounding ECM, promoting both vascular network development and tumour growth^34^. Lei Tang et al. used an integrated model to analyse the delivery of chemotherapeutic drugs in three-dimensional space, showing that drugs are more likely to diffuse into low-pressure regions^35^. From these models, several angiogenesis-related factors were identified which either promote or inhibit angiogenesis in the microenvironment. However, how the identified pro- and anti-angiogenic factors and their receptors regulate angiogenesis is not well understood.

The angiopoietin family consists of four types of angiogenic factors, Ang1 to Ang4, which are expressed by vascular endothelial cells. These factors regulate adhesion between vascular wall cells and endothelial cells^36^. Ang1 promotes endothelial–parietal cell adhesion and vascular maturation by binding to the receptor tyrosine kinase Tie-2. On the other hand, Ang2 is secreted under a hypoxic state and acts as an antagonist of the Tie-2 receptor to weaken cell–cell adhesion. These two factors regulate the stabilization and remodelling of blood vessels and capillary sprouting in the early stage of angiogenesis^37^. Other molecules also regulate angiogenesis such as platelet-derived growth factor (PDGF) that affects blood vessel maturation, and fibroblast growth factor (FGF) that indirectly promotes angiogenesis by stimulating blood vessel receptors (FGFR)^38^. The kinetics of angiogenic growth depends on the balance between these promoting and suppressing factors. Thus, the length of new vessels is not only characterized by monotonical growth but also by regression and growth^39^. The inhibition of VEGF receptor signalling induces regression of angiogenesis, while its regrowth has been observed after inhibition withdrawal^40^. These phenomena have been mathematically proven in a VEGF receptor inhibition study^41^. However, to the best of our knowledge, no mathematical model has reproduced the mechanism of vascular regression that occurs without drug administration.

This study aimed to develop a three-dimensional simulation model integrating angiogenesis and tumour growth. Temporal vascular regression was reproduced by incorporating the regulatory function of vascular flexibility using angiopoietin. Finally, an administration simulation of the drug, bevacizumab was conducted, during which the dose and timing of drug administration were considered to maximize its therapeutic effect.

## Methods

### Existing models

In this study, we modified the mathematical model of Lei Tang et al.^35^. Briefly, the model simulated the physical and chemical processes of initial tumour growth, from an un-vascularized state to a vascularized state, in three-dimensional space. The distribution of each factor, such as oxygen (O_2_) and TAF, was described using partial differential equations and discretised and solved numerically.

The model reproduced tumour microenvironment pressure by combining cell-induced tumour pressure (CTP) and vascular perfusion-induced tumour pressure (VTP). CTP at spatial point ${X}_{0}$(${x}_{0}, {y}_{0}, {z}_{0}$)was calculated as the sum of pressures caused by N tumour cells.

1 $${{p}^{c}}_{{X}_{0}}=\sum_{i=1}^{N}{{p}^{c}}_{({X}_{0},{X}_{i})},$$

2 $$\mathrm{N}={(2\mathrm{k}+1)}^{3},$$

where ${{p}^{c}}_{{X}_{0}}$ is the tumour pressure that point ${X}_{0}$ receives from surrounding tumour cells, and ${p}^{c}$ is the tumour pressure that point ${X}_{i}$ gives to point ${X}_{0}$. N, calculated in Eq. (2), is the number of all grid points contained in a cube consisting of k grid points per side centred on the point ${X}_{0}$. The pressure was added to point ${X}_{0}$ only if there were tumour cells at each point. In vascularized tumours, VTP was calculated by the sum of pressures caused by vascular endothelial cells present next to *k* point ${X}_{0}$, using a method similar to CTP [Eq. (1)]. Pressure p was determined by adding the CTP and the VTP in each grid point. Tumours were more likely to grow in the direction of lower pressure p.

O_2_ was assumed as a necessary nutrient for tumour growth. Using Eq. (3) below, four processes of O_2_ kinetics were calculated, including diffusion, convection, O_2_ secretion from blood cells, and O_2_ consumption by tumour cells.

3 $$\frac{\partial \mathrm{n}}{\partial \mathrm{t}}={D}_{n}{\nabla }^{2}\mathrm{n}-\nabla \cdot \left(\mathbf{u}\cdot \mathrm{n}\right)+{\rho }_{n}\left({r}_{V},\left({p}_{V}-p\right)\right){\delta }_{{\Sigma }_{V}}-{\lambda }_{n}\left({A}_{i}\right){\delta }_{{\Omega }_{T}},$$

where n is the O_2_ concentration, ${D}_{n}$ is the diffusion constant of O_2_, **u** is the pressure gradient, ${A}_{i}$ is the cellular activity of tumour cells, and ${r}_{V}$ means removal term. ${\delta }_{{\Sigma }_{V}}$ indicates that the activity occurs in all vascular cells, while ${\delta }_{{\Omega }_{T}}$ indicates that it occurs in all tumour cells. The third term of Eq. (3) describes the kinetics of O_2_ secretion from blood cells and is defined by the following Eq. (4):

4 $${\rho }_{n}\left({r}_{V},\left({p}_{V}-p\right)\right)={\rho }_{n0}{R}_{i}\mathrm{W},$$

5 $${\text{W}} = \left\{ {\begin{array}{*{20}l} {\frac{{p_{V} - p}}{{p_{V} }},} \hfill & {(p_{V} - p > 0)} \hfill \\ {0,} \hfill & {\left( {p_{V} - p \le 0} \right)} \hfill \\ \end{array} } \right.,$$

where ${\rho }_{n0}$ is the O_2_ supply rate and ${R}_{i}$ is the radius of the vessel, as described by Eq. (12). Equation (5) defines the pressure gradient W of the arterial wall, which was calculated from the ratio of arterial pressure ${p}_{V}$ to the pressure p of each grid points. Thus, the actual rate of O_2_ supply ${\rho }_{n}$ was determined by multiplying ${\rho }_{n0}$ by a value corresponding to the arterial radius and the size of the pressure gradient. If the value of n was less than 0 in these calculations, n is set to 0.

The rate of O_2_ consumption, shown in the fourth term of Eq. (3), was determined using Eq. (6).

6 $${\lambda }_{n}\left({A}_{i}\right) ={\lambda }_{n0}{A}_{i},$$

7 $${A}_{i}=\frac{n}{n+1}\mathrm{exp}\left(-5{\left(w-1\right)}^{4}\right),$$

where ${\lambda }_{n0}$ represents the O_2_ consumption rate and w is the concentration of carbon dioxide (CO_2_). In Eq. (7), cell activity ${A}_{i}$ determines the activity status of each tumour cell. It was calculated according to the nutrient acquisition status estimated from the [O_2_] ([X] indicates the concentration of X) and [CO_2_] in each tumour cell. Tumour cell status classification was set as active when cell activity, ${A}_{i},$ ≥ 0.5, and quiescent when ${A}_{i}$ < 0.5. The change between active and quiescent states was reversible.

In addition to cell activity, Lei Tang et al.^35^ introduced a new concept, Cell Vital Energy (CVE), the energy stored in the cell for proliferation. This concept explained tumour cell progression to proliferation, as well as their life and death. CVE is denoted by ${V}_{i}$ and it changes depending on the cell activity ${A}_{i}$ of each tumour cell. ${V}_{i}$ is defined as below,

8 $$\frac{{dV_{i} }}{{dt}} = \left\{ {\begin{array}{*{20}l} {\frac{{A_{i} }}{{A_{i} + 1}}{\text{k}}_{{{\text{active}}}} } \hfill & {(active)} \hfill \\ { - {\text{k}}_{{{\text{quiescent}}}} } \hfill & {(quiescent)} \hfill \\ \end{array} } \right.,$$

where ${\mathrm{k}}_{\mathrm{active}}$ and ${\mathrm{k}}_{\mathrm{quiescent}}$ are constants representing the rate of increase for CVE inactive tumour cells and quiescent cells, respectively. In the active state (${A}_{i}$ ≥ 0.5), tumour cells actively store energy, CVE, for their proliferation, while in the quiescent state (${A}_{i}$ < 0.5), tumour cells consume CVE to support cellular life, ${k}_{quiescent}$. In this process, tumour cell proliferation begins when CVE exceeds the threshold level, t_CVE_ and the cell changed irreversibly to the necrotic state when CVE ≤ 0,

Although there are many types of molecules as TAFs, Lei Tang et al. implemented them as a single element that promotes capillary sprouting^35^. This element of TAF is treated as a VEGF in our model because VEGF is considered to be the most principal factor of TAF^8^. They hypothesized that quiescent tumour cells induced by hypoxia would secrete VEGF, subsequently stimulating blood vessels and causing capillary sprouting. The VEGF concentration, c, was described using the following partial differential Eq. (9):

9 $$\frac{\partial \mathrm{c}}{\partial \mathrm{t}}={D}_{c}{\nabla }^{2}\mathrm{c}-\nabla \cdot \left(\mathbf{u}\cdot \mathrm{c}\right)+{\rho }_{c}\left(n\right){\delta }_{{\Omega }_{T}}-{\lambda }_{c}\left({r}_{V}\right){\delta }_{{\Sigma }_{TEC},}$$

where ${D}_{c}$ is the VEGF diffusion constant and ${\delta }_{{\Sigma }_{TEC}}$ indicates occurrence in tip endothelial cells (TEC). Each term describes VEGF diffusion, convection, secretion by tumour cells, and removal from the vessel. TEC is the apical part of a vessel and migrates according to the [VEGF] gradient^42^. The rate of VEGF secretion ${\rho }_{c}\left(n\right)$, and the rate of VEGF consumption ${\lambda }_{c}\left({r}_{V}\right)$, were assumed to be proportional to O_2_ concentration, n, and vessel radius, ${R}_{i}$, respectively.

10 $${\rho }_{c}\left(n\right)={\rho }_{c0}\left(1-\mathrm{n}\right),$$

11 $${\lambda }_{c}\left({r}_{V}\right)={\lambda }_{c0}{R}_{i},$$

12 $${R}_{i}=\frac{{Age}_{i}}{{Age}_{i}+{k}_{AR2}}{k}_{AR1},$$

where ${\rho }_{c0}$ is the secretion rate of VEGF, ${\lambda }_{c0}$ is the consumption rate of VEGF, ${Age}_{i}$ is the time elapsed since the sprouting of new vessels, and ${k}_{AR1}$ and ${k}_{AR2}$ are constants. The higher the level of hypoxia, the higher the rate of VEGF secreted [Eq. (10)]. Equation (12) indicates that the vascular radius, ${R}_{i}$, grows faster soon after capillary sprouting. As the vascular radius increases with time, the ability to both deliver nutrients to tumour cells and remove metabolic wastes increases. The formula for [CO_2_], w is the same as the one reported by Lei Tang et al. The process of CO_2_ kinetics was calculated, including diffusion, convection, secretion by tumour cells, and removal by blood cells in a similar manner as that used for VEGF. These were modelled via partial differential equations.

To determine the direction and velocity of vessel extension, Lei Tang et al.^35^ hypothesized that vessels were likely to extend to higher [VEGF]. TEC at a point $${X}_{i}({x}_{i}$$, $${y}_{i}$$, $${z}_{i})$$ could extend in any of the six directions represented as directional derivatives at a point $${X}_{i}({x}_{i}$$, $${y}_{i}$$, $${z}_{i}$$). The cycle $$\uptau ({x}_{i}, {y}_{i}, {z}_{i}),$$ which is the rate of vascular proliferation required for endothelial cell proliferation, was determined using inner and outer pressure differences of tip endothelial cells [see Eq. (13)]^43^.

13 $$\uptau ({x}_{i}, {y}_{i}, {z}_{i})={k}_{v}{\left({\alpha }_{v}\right)}^{\Delta p({x}_{i}, {y}_{i}, {z}_{i})},$$

where parameters ${k}_{v}$ and ${\alpha }_{v}$ maybe adjusted to the desired arterial velocity, and $\Delta p({x}_{i}, {y}_{i}, {z}_{i})$ is the difference in pressure between the inside and outside of the vessel wall. The rate of vessel extension varied through pressure differences calculated using Eq. (13).

In this study, we used the same method as that used by Lei Tang et al.^35^ unless specified otherwise.

### Implementation of angiopoietin

This section describes our modifications. Angiopoietin was added to the model as a capillary sprouting angiogenesis factor. The angiopoietin family regulates adhesion between vessel wall cells and endothelial cells by binding to receptor-type tyrosine kinase Tie-2, which is expressed by vascular endothelial cells and regulates angiogenesis^36^. Only two models incorporated the expression balance of angiopoietins into capillary sprouting conditions, while no individual cell states were modelled^44,45^.

Here we incorporated Ang1, which stimulates Tie-2 on vascular endothelial cells during angiogenesis, and Ang2, which inhibits Ang1 binding to Tie-2^37^, into our model. The balance of these components changes the vascular state. Equations (14) and (15) showing the distribution of Ang1 concentration, ${a}_{1}$, and Ang2 concentration, ${a}_{2}$, were derived using diffusion, convection, secretion, and elimination terms, in a similar way as that for VEGF and O_2_, from the Lei Tang et al. model^35^.

14 $$\frac{\partial {a}_{1}}{\partial \mathrm{t}}={D}_{{a}_{1}}{\nabla }^{2}{a}_{1}-\nabla \cdot \left(\mathbf{u}\cdot {a}_{1}\right)+{\rho }_{{a}_{1}}\left({\theta }^{EC}\right){\delta }_{{\Sigma }_{EC}}-{\lambda }_{{a}_{1}}\left({a}_{1}\right){\delta }_{{\Sigma }_{EC}},$$

15 $$\frac{\partial {a}_{2}}{\partial \mathrm{t}}={D}_{{a}_{2}}{\nabla }^{2}{a}_{2}-\nabla \cdot \left(\mathbf{u}\cdot {a}_{2}\right)+{\rho }_{{a}_{2}}\left({\theta }^{TEC}\right){\delta }_{{\Sigma }_{TEC}}-{\lambda }_{{a}_{2}}\left({a}_{2}\right){\delta }_{{\Sigma }_{V},}$$

where ${D}_{{a}_{1}}$ and ${D}_{{a}_{2}}$ are Ang1 and Ang2 diffusion coefficients, respectively, ${\theta }^{EC}$ is endothelial cell density, ${\theta }^{TEC}$ is the density of TEC in a new vessel, and ${\delta }_{{\Sigma }_{EC}}$ indicates occurrence in endothelial cells. Angiopoietins act in a paracrine fashion, in which a substance secreted by a specific cell exerts local actions around itself via tissue fluid, without passing through the blood^46^. Diffusion coefficient values ${D}_{{a}_{1}}$ and ${D}_{{a}_{2}}$ were established to reproduce the nature of angiopoietin, which is mainly secreted by vascular endothelial cells to act on surrounding endothelial cells^47^. The rate of angiopoietin secretion and consumption was expressed via the following Eqs. (16) to (21):

16 $${\rho }_{{a}_{1}}\left({\theta }^{EC}\right)={\rho }_{{a}_{1\_0}}{\theta }^{EC},$$

17 $${\rho }_{{a}_{2}}\left({\theta }^{TEC}\right)={\rho }_{{a}_{2\_0}}{\theta }^{TEC},$$

18 $${\theta }^{EC}=\frac{{EC}_{N}}{{(2k+1)}^{3}},$$

19 $${\theta }^{TEC}=\frac{{TEC}_{N}}{{(2k+1)}^{3}},$$

20 $${\lambda }_{{a}_{1}}\left({a}_{1}\right)={\lambda }_{{{a}_{1}}_{0}}\left({a}_{1}-1\right),$$

21 $${\lambda }_{{a}_{2}}\left({a}_{2}\right)={\lambda }_{{a}_{2}\_0}{a}_{2},$$

where ${\rho }_{{a}_{1\_0}}$ and ${\rho }_{{a}_{2\_0}}$ are Ang1 and Ang2 generation rates, respectively, ${EC}_{N}$ and ${TEC}_{N}$ are endothelial cells and TECs in the N grid points centred on each point, respectively, and ${\lambda }_{{a}_{1}\_0}$ and ${\lambda }_{{a}_{2}\_0}$ are Ang1 and Ang2 consumption rates, respectively. Angiopoietin diffusion and secretion rate values were obtained from a model by Trachette Jackson et al.^48^.

The secretion rate of angiopoietin was assumed to increase with density of endothelial cells, or TECs, in each grid point. Endothelial cell density was calculated at all grid points ${(2\mathrm{k}+1)}^{3}$ of a cube consisting of one side (2k + 1) grid points centred on each point. The consumption rate was determined via the concentration of angiopoietin, itself, in the surroundings.

**Figure 1:**
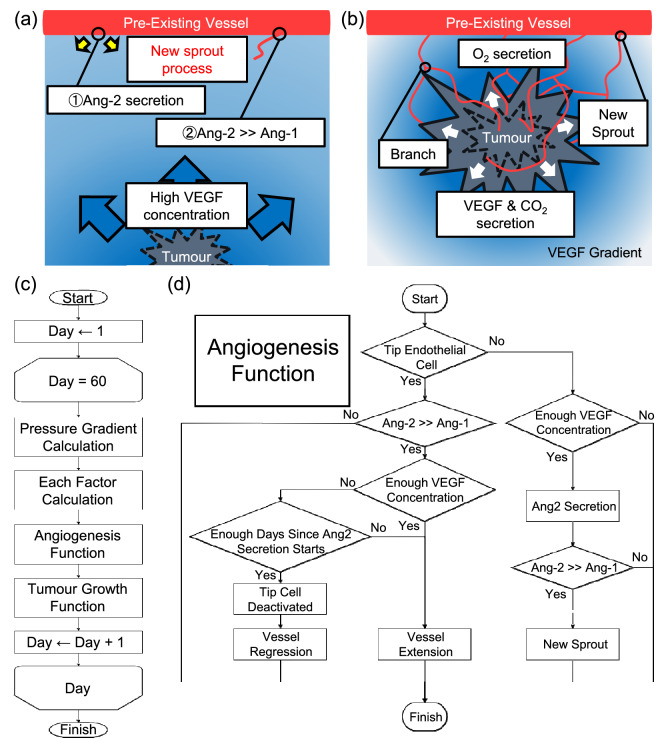
Model concept and flowchart. (**a**) and (**b**) Formation of a new vascular network via angiogenesis: Existing vascular endothelial cells secrete Ang2 in response to tumour-secreted VEGF stimulation. When [Ang2] levels become sufficiently higher than [Ang1] levels, the vessel releases wall cells and new vessels sprout. Sprouting vessels extend according to the [VEGF] gradient. Neovascularization forms a dense vascular network around the tumour. Tumours proliferate utilizing a well-developed vascular network which allows them to acquire nutrients and expel metabolites; (**c**) An overall flowchart of the simulation. The pipeline, including pressure gradient, each factor, angiogenesis, and tumour growth, was repeated for 60 days; (**d**) Details of angiogenesis function. VEGF stimulation caused Ang2 secretion in blood vessels, resulting in [Ang2] >> [Ang1] and the extension of new blood vessels. Tip endothelial cells in [Ang2] > > [Ang1] are deactivated and regressed, with probability, ${p}_{rg}$, calculated from [VEGF].

We described capillary sprouting and regression. In the model by Lei Tang et al., only the effect of [TAF] was considered as a promoting factor for the sprouting of new blood vessels; however, in the current study, capillary sprouting and branching were also determined via the distance of each cell from the tumour and the balance of angiopoietin concentration.

22 $${p}_{BH}={k}_{BH}{({\alpha }_{BH})}^{log\left({c}_{i}\right)},$$

23 $${k}_{BH}={\left(\frac{{l}_{(T,{X}_{i})}}{{l}_{max}}\right)}^{f}{k}_{BH\_0},$$

where ${c}_{i}$ is the concentration of VEGF at the point i, ${p}_{BH}$ is the probability of capillary sprouting or branching at each point, ${k}_{BH}$ is the function of the probability of sprouts, and ${\alpha }_{BH}$, ${k}_{BH\_0}$, f and h are the adjusted constants. The constant ${k}_{BH\_0}$ was higher than ${k}_{BH}$ in the model proposed by Lei Tang et al. because it was adjusted to obtain a uniform sprouts throughout the simulation space and to facilitate comparison under various conditions [Eq. (23)]. ${k}_{BH}$ is a simple constant in the original model, but we multiplied the constant ${k}_{BH\_0}$ by ${l}_{(T,{X}_{i})}$, which is the distance from the centre of the initial tumour to each cell. Figure 1a,b shows the capillary sprouting process in this simulation. Capillary sprouting probability ${p}_{BH}$ was calculated repeatedly during the simulation and, as [VEGF] changed, ${p}_{BH}$ also changed. These calculations determined new branching points and initiated Ang2 secretion.

Equation (24) reflects the conditions under which the balance of angiopoietin concentration causes an unstable blood vessel state similar to the conditions used by Plank et al.^45^ When this condition was met, a new vessel could begin to sprout.

24 $${a}_{2}>h {a}_{1},$$

In this study, we also reproduced the mechanism of vascular regression. Vascular regression has been reported in clinical trials and is said to affect the rapid growth of neovascularization^39^. The conditions for vessels to regress were established in the same way as in Eq. (22).

25 $${p}_{rg}={k}_{rg}{({\alpha }_{rg})}^{log\left({c}_{i}\right)},$$

26 $${k}_{rg}={\left(1-\frac{{l}_{(T,{X}_{i})}}{{l}_{max}}\right)}^{g}{k}_{rg\_0},$$

where ${p}_{rg}$ is the regression probability, ${k}_{rg}$ is the function of the probability of regression, and ${\alpha }_{rg}$, ${k}_{rg\_0}$, and $g$ are the constants. When the TEC satisfied the condition of Eq. (24), vascular regression started with the probability calculated by Eq. (25). It was reported that neovascularization cannot retain its shape after prolonged exposure to Ang2 by clinical experimentation^36,37^, so vessel regression starts a certain period after the sprouting of new vessels in this simulation. Detailed conditions are described at assumption 7) in the section “Analysis conditions and assumptions”.

### Anti-angiogenic therapy

This section describes our model for anti-angiogenic therapy. We simulated the administration of Avastin, an anti-angiogenic drug. Avastin binds to VEGF and inhibits the sprouting of new blood vessels. We formulated the following Eqs. (27) and (28) to reproduce this function. Each term describes diffusion, convection, Avastin secretion from blood cells, Avastin consumption by binding to VEGF, and decay.

27 $$\frac{\partial \mathrm{d}}{\partial \mathrm{t}}={D}_{d}{\nabla }^{2}\mathrm{d}-\nabla \cdot \left(\mathbf{u}\cdot \mathrm{d}\right)+{\rho }_{d}\left({r}_{V},\left({p}_{V}-p\right), dv(t)\right){\delta }_{{\Sigma }_{V}}-B-{\lambda }_{d}d$$

28 $$B=d c,$$

where d is [Avastin], ${D}_{d}$ is the Avastin diffusion constant, and ${\lambda }_{d}$ is a decay rate of Avastin. Here, the term for Avastin secretion is the same as that used in the drug administration model of Lei Tang et al. Equation (28) shows the calculation of B, which is the amount of binding of Avastin to VEGF. If B is greater than d or c, it was adjusted to one of the maximum values. When the drug administration simulation was performed, − B was added to the VEGF calculation Eq. (9).

### Analysis conditions and assumptions

The simulation environment consisted of a 1 cm^3^ 3-dimensional space divided into 200 × 200 × 200 points on a grid. At the beginning of the simulation, five tumour cells were placed near the centre of this virtual space. Each lattice point included a maximum of one tumour cell or vascular endothelial cell.

As a numerical calculation, each partial differential equation was normalized and solved with Finite Difference Method. For dimensionless parameters, we employed several parameters used in the model of Jackson et al.^48^, including the density of endothelial cells e0, the concentration of VEGF at the tumour boundary c0, and the concentration of Ang-1 a0. The boundary conditions were set as follows: n = 1, w = 0, c = 0, d = 0, ${a}_{1}$ = 1, and ${a}_{2}$ = 0. This tumour angiogenesis simulation was conducted for 60 days with 33 steps per day.

Initial assumptions included O_2_ and Ang1 uniformly diffused throughout the space, sufficient nutrients present, and blood vessels in a stable state. The assumptions were as follows:

1. Tumour cells become active when cell activity, ${A}_{i}$, is ≥ 0.5 and quiescent when ${A}_{i}$ < 0.5. These changes are reversible.
2. Tumour cells irreversibly change from a survival state to a necrotic state when CVE is < 0.
3. Tumour pressure, ${p}_{0}$, = 60 [mmHg] and capillary pressure, ${p}_{V}$, = 30 [mmHg].
4. Tumours migrate or grow toward the lower interstitial pressure of 26 neighbouring grid points.
5. Ang2 and Ang1 are expressed in TEC and normal endothelial cells, respectively.
6. Ang2 expression begins when an existing vessel satisfies the condition of Eq. (22) upon VEGF stimulation. Subsequently, new sprouts occur when the condition of Eq. (24) is satisfied.
7. More than 3 days after new sprouts, vascular regression occurs with the probability calculated by Eq. (25) when the condition of Eq. (24) is satisfied. Even if one of the blood vessels in the two halves regresses, the other continues to grow in response to [VEGF].

The various parameters used in this simulation are shown in Table S1. These parameters were used unless specified otherwise. Figure 1c,d shows a flowchart of the entire program, as well as a detailed flowchart of capillary sprouting and regression. A tumour growth flowchart is available in Supplemental Information (Figure S1). All simulations described in this manuscript were performed on an INTEL XEION CPU E3-1230 V2 3.30 GHz, RAM 20 GB memory computer and using MATLAB R2019B (MATHWORKS, Natick, MA, USA) software. Source code is available upon request.

## Results and discussion

**Figure 2:**
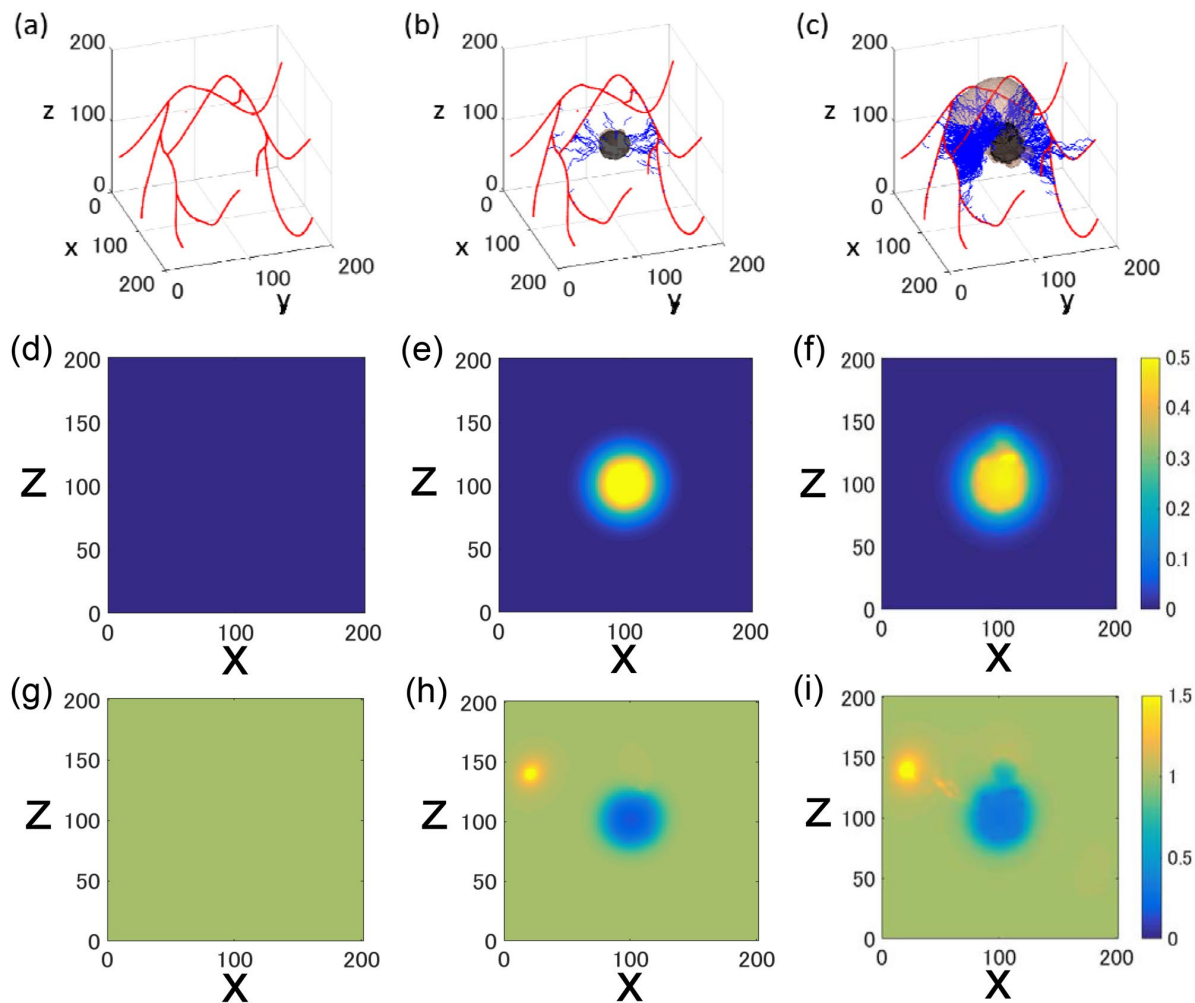
Snapshot of the spatial distribution of blood vessels, tumours, and related factors. Snapshots on day 0 (**a**, **d**, and **g**), 40 (**b**, **e**, and **h**), and 60 (**c**, **f**, and **i**). Panels (**a**–**c**) show vascular and tumour status. Red and blue curves show existing blood vessels and the newly sprouted vessels, respectively. Initially, we placed five tumours in the central part of the virtual space; (**d**–**f**) [VEGF] at y = 100. VEGF was secreted by tumour cells in the hypoxic state. Additionally, VEGF was not present in the initial phase; (**g**-**i**) Time evolution of [O_2_] at y = 100. Oxygen was uniformly present throughout the initial phase. Oxygen was secreted by blood vessels and consumed by tumour cells.

We performed a tumour angiogenesis simulation for 60 days. Figure 2a shows the initial existing vasculature. Over time, various factors, such as VEGF, nutrition (O_2_), metabolic waste (CO_2_), and angiopoietins, were repeatedly calculated. Movies of angiogenesis-induced vascular network formation and tumour growth, and VEGF diffusion, are available in Video S1 and Video S2. According to the time transition of 60 days in Video S1, the density of new blood vessels increased up to the middle of the entire period. Then, it started to decrease, and it increased again after a certain time.

### Spatial distribution of tumour microenvironment

Figure 2a–c shows a snapshot of the vessel and tumour spatial distribution at days 0, 40, and 60. Red and blue curves represent existing vessels and new vessels, respectively. Figure 2d–f shows the x–z diagram of [VEGF] distribution at days 0, 40, and 60, at y = 100. The entire space in Fig. 2d is blue, indicating the absence of VEGF. On days 40 and 60, the yellow area which indicates high [VEGF], gradually spreads from the central area, where the tumour was initially present (x = 100, y = 100, z = 100). Compared with Fig. 2a–c, new vessels also proliferated in high [VEGF] yellow areas.

Figure 2g–i shows the [O_2_] distribution. Initially, the whole space is green, indicating the uniform distribution of O_2_ in the space (Fig. 2g). On day 40, the tumour area was blue, indicating that O_2_ was consumed (x = 100, y = 100). Furthermore, existing vessels turned yellow (x = 20, z = 140), indicating that O_2_ was secreted and highly concentrated (Fig. 2h). On day 60, following tumour neovascularization, areas around new vessels also turned yellow, implying local O_2_ secretion for tumour growth (Fig. 2i). CO_2_ was generated in the central part of the tumour in the same way as VEGF (x = 100, y = 100, z = 100), confirming nutritional status deterioration inside the tumour (Figures S2 and S3). In tumours, O_2_ is generally consumed and VEGF is secreted, under hypoxic conditions^5^. Figure 2d–i shows that the central tumour area was hypoxic and high in VEGF.

### Vessel length growth

**Figure 3:**
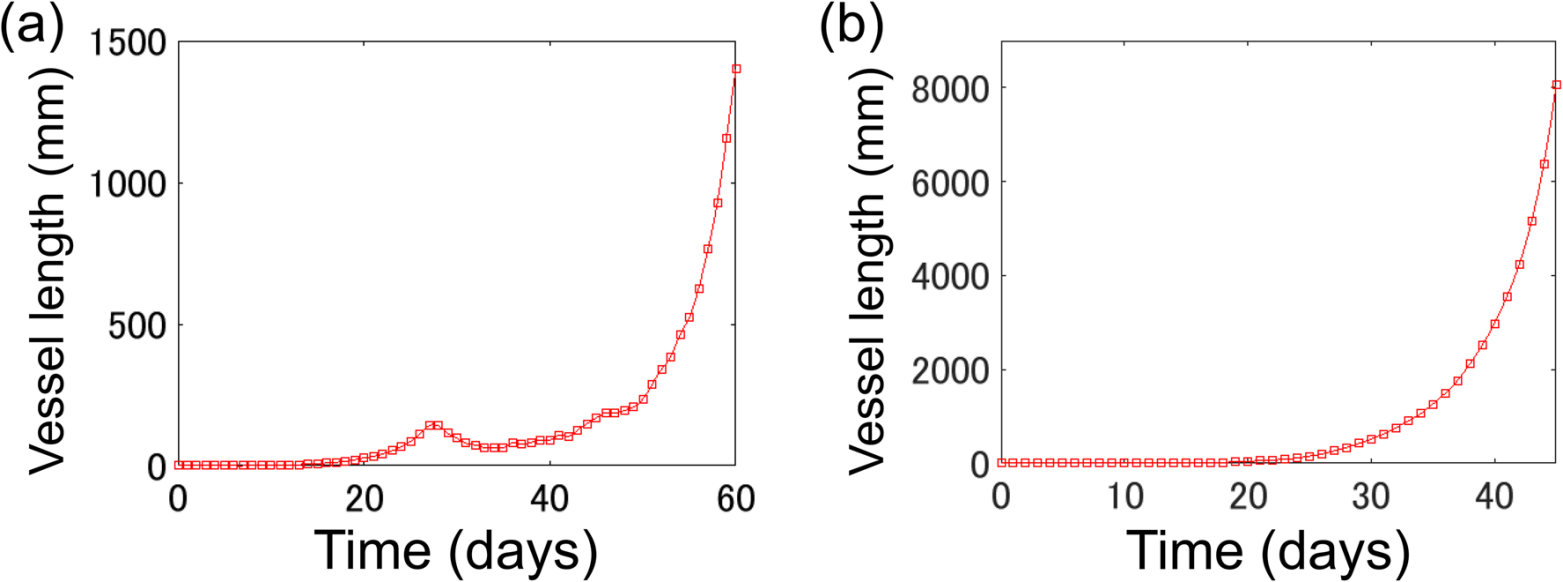
Time-course of new vessel length. (**a**) Changes in the length of new vessels during the 60-day tumour angiogenesis simulation. (**b**) Changes in the length of new vessels during the 60 day tumour angiogenesis simulation, in the absence of angiopoietin-induced regression. (**a**,**b**) The x- and y-axes show time and length, respectively, of new vessel length.

**Figure 4:**
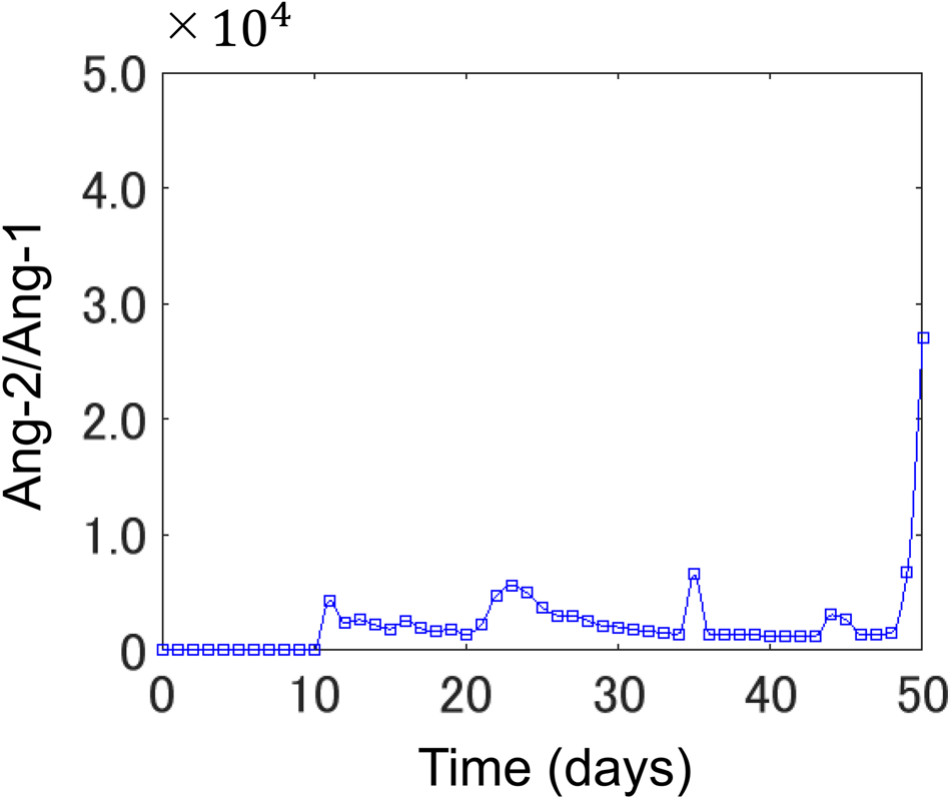
Ang2/Ang1 in the new vessel sprouting area. The x-axis represents time, while the y-axis represents the average Ang2/Ang1 values at new blood vessel branching points.

Figure 3 shows a temporal change in the total length of newly generated vessels; indicated in blue in Fig. 2a–c. Figure 3a,b shows simulation results with and without angiopoietin. When angiopoietin was implemented, the length of new vessels increased monotonically until $1.41\times {10}^{2}$ mm on day 27, from 0 mm on day 0 (Fig. 3a); then showing a decrease to $6.32\times {10}^{1}$ mm, before again increasing rapidly from day 35 (Fig. 3a). Three phases in angiogenesis can be reproduced here: proliferative, regressive, and re-proliferative. In the absence of angiopoietin, the length of new vessels continued to increase monotonically throughout the simulation (Fig. 3b), reaching $8.06\times {10}^{3}$ mm on day 45; 46.7 times longer than that achieved with angiopoietin. Figure 3b shows changes in vascular length until day 45 for ease of comparison. The length of new vessels at day 60 was 2.1 $\times {10}^{5}$. Figure 4 shows the time-series change in the average of angiopoietin concentration balance, Ang2 /Ang1, at all grid points where new blood vessels sprout (Figure S4). Initially, Ang2/Ang1 values are close to zero because of the Ang1 >> Ang2 condition, however, on day 10, this ratio begins to increase, continuing to rise until day 20, at which point there is no change for some time, before again rapidly increasing from day 50. Since the value is large after the 50th day, the graph presents data until day 50 for visibility. The Ang2/Ang1 value at day 60 was 1.81 $\times {10}^{5}$.

In the growth of new vessels (Fig. 3a), we infer why the three aforementioned phases occurred. From the beginning of the simulation to the proliferative phase, [VEGF] gradually increased, especially in tumours with a central location (Figure S5). As shown in Eq. (22), the probability of capillary sprouting increased with increases in [VEGF]. As Ang2 secretion commenced in the sprouting grid points, Ang2/Ang1 began to increase on day 10, reaching a temporary peak on day 23 (Fig. 4). Late to the increase in Ang2/Ang1, the length of new vessels began to increase on day 15, suggesting that increased [Ang2], compared to [Ang1], was an important condition for sprouting of new vessels (Fig. 3a). New vessel length entered a regressive phase following a temporary peak in Ang2/Ang1 values on day 27. In addition to TEC increases satisfying the vascular destabilization condition indicated in Eq. (24), sufficient time had passed since capillary sprouting, resulting in increased vascular regression and decreased total new vessel length. The vessel entered a re-proliferative phase on day 35. In this phase, [VEGF] secreted by tumour cells had diffused throughout the simulation space, compared to when vascular regression began (Figure S5). With this increase in [VEGF], the probability of angiopoietin-induced vascular regression mechanisms decreased, and the length of new vessels began to increase again.

As shown by the above process, Ang2 has been previously speculated to act as an Ang1 antagonist, generating angiogenesis, and vascular regression^36^. In this study, we were able to quantitatively describe the mechanism of angiopoietin-induced vascular regulation. A series of these phenomena, such as the temporary decrease of blood vessels and the occurrence of rapid re-proliferation, have also been reported in several previous clinical experiments, suggesting that the actual phenomena can be reproduced in this simulation^39,49^. Therefore, regulation of vessel wall cell release and adhesion by a balance of angiopoietin concentration, and VEGF stimulation may regulate changes in vessel length.

### Relationship between timing of drug administration and tumour growth

**Figure 5:**
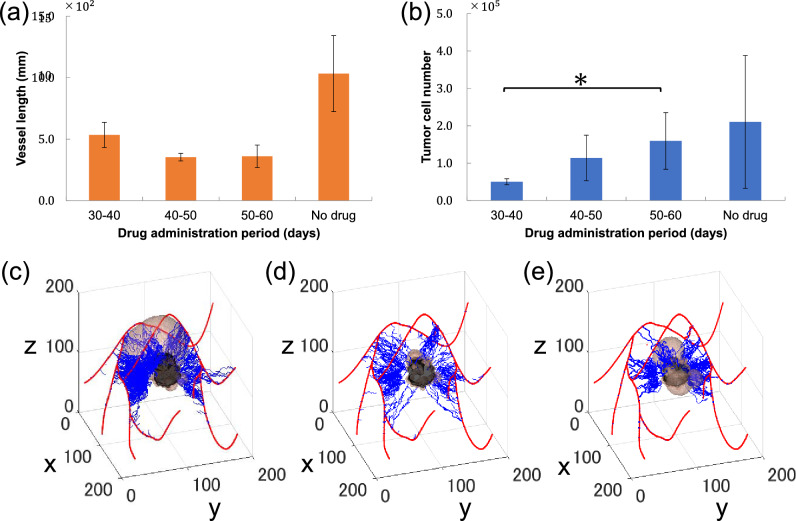
Change in simulation results with timing of administration. (**a**,**b**) Changes in tumour number and neovascular length on day 60, according to the time of administration: The x-axis represents drug administration conditions, while the y-axes represent the number of tumour cells (**a**) and the length of new vessels (**b**) on day 60. Bars and error bars show the mean and standard deviation, respectively, for each medication condition. *P < 0.05 (Student’s *t*-test, Bonferroni correction (n = 5)). (**c**–**e**) Vascular and tumour status on day 60 in the absence of the drug, and with drug administration for 30–40 and 50–60 days: The red curve shows existing blood vessels in the human body, while the blue curve shows newly sprouted vessels.

We observed the effect of administered bevacizumab on tumour growth and angiogenesis. Initially, to estimate the most effective administration time for tumour growth inhibition, we performed Avastin-treatment experiments in three different administration periods: 30–40 days, 40–50 days, and 50–60 days, in a 60-day simulation. Figure 5a shows the length of new vessels at the end of the simulation for each condition. The shortest final vessel length was observed when the drug was administered for 50–60 days, with a mean vessel length and standard deviation (SD) of 3.60 $\times {10}^{2}$ ± 0.914 mm $\times {10}^{2}$. On the other hand, the longest total length of new vessels was observed when the drug was administered for 30–40 days, with a mean vessel length and SD of 5.34 $\times {10}^{2}$ ± 1.03 mm$\times {10}^{2}$. Although administration for 50–60 days resulted in 0.887 times longer vessel length than that seen after administration for 30–40 days, *P* = 0.0669 (Student's *t*-test, Bonferroni correction), it was not statistically significant. Figure 5b shows the number of tumour cells at the end of the simulation for each condition. The lowest final number of tumour cells was observed when the drug was administered for 30–40 days, with a mean number of tumour cells and SD of 0.503 $\times {10}^{5}$ ± 0.0794 $\times {10}^{5}$. On the other hand, the highest number of tumour cells was observed when the drug was administered for 50–60 days, with a mean number of tumour cells and SD of 1.59 $\times {10}^{5}$ ± 0.759 $\times {10}^{5}$. Administration for 50–60 days resulted in 3.16 times as many tumour cells as those observed after administration for 30–40 days, with *P* = 0.0379 (Student's *t*-test corrected Bonferroni correction) confirming statistical significance. These results indicate an increase in new vessel length and the number of tumour cells with early and late drug administration, respectively. Figure 5c–e shows vascular and tumour status at day 60 without drug administration, and with drug administration for 30–40 and 50–60 days.

Both final new vessel length and tumour cell numbers were found to be shorter and larger, respectively, with later bevacizumab administration. New vessel length changes in the non-drug condition indicated that length began to increase from day 35, increasing faster from 50–60 days than before day 50 (Fig. 3a). From these results, we formulated the following hypothesis. First, the final length of new vessels was smallest when the drug was administered for 50–60 days because VEGF function was suppressed during the period when the vessel increase rate was the most intense, minimizing the occurrence of capillary sprouting. However, delayed drug administration did not inhibit angiogenesis in the initial stages, with tumour cells acquiring a new vascular network. Tumour cells then acquired nutrients from this vascular network and grew large enough to acquire nutrients from existing vessels. Thus, even if bevacizumab could be administered to reduce newly sprouted vessels, its effect on tumour reduction would be diluted. This can be deduced from the fact that tumour cells (Fig. 5e), which were administered bevacizumab for 50–60 days, had more areas where the tumour was close to existing vessels than those in Fig. 5d (e.g., x = 30, y = 130, z = 120). Therefore, we concluded that bevacizumab most effectively inhibited initial cancerous tumour growth when administered before tumour cells were large enough to acquire nutrients from existing blood vessels.

### Relationship between intervals of drug administration and tumour growth

**Figure 6:**
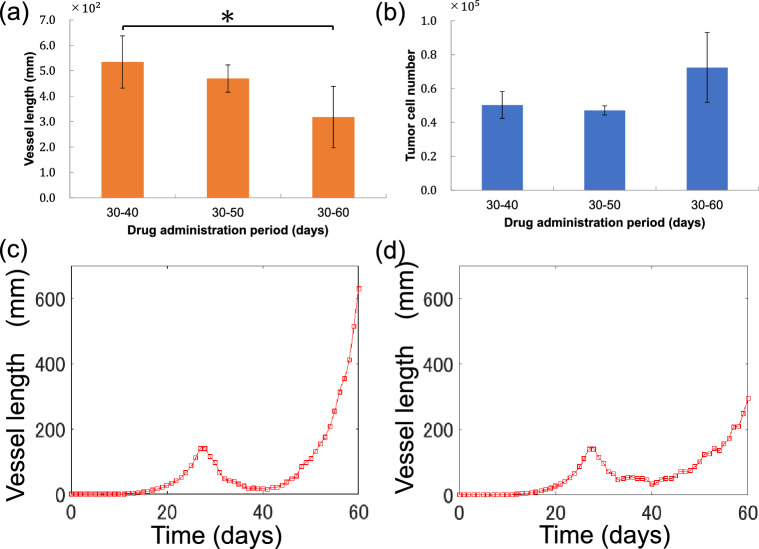
Change in simulation results with intervals of administration (**a**,**b**) Changes in tumour number and length of new vessels on day 60, according to the treatment period: The x-axis represents treatment conditions, while the y-axes represent the number of tumour cells (**a**) and the length of new vessels (**b**) on day 60. Brs and error bars show the mean and standard deviation, respectively, for each medication condition. *P < 0.05 (Student's *t*-test, Bonferroni correction (n = 5)). (**c**,**d**) Changes in new vessel length during 30–40 days and 30–60 days of drug treatment: The x-axis shows time, the y-axis shows length of new vessels.

We investigated optimal anti-angiogenic agent administration intervals to make treatment more effective. Figure 6a shows the total length of new vessels at the end of the simulation in each condition. The shortest total length of new vessels was observed when the drug was administered for 30–60 days, with a mean vessel length and SD of 3.18 $\times {10}^{2}$ ± 1.21 mm $\times {10}^{2}$. On the other hand, the longest total length of new vessels was observed when the drug was administered for 30–40 days. The effect was 0.594 times longer after administration for 30–60 days than that for 30–40 days, with *P* = 0.0472 (Student's *t*-test, Bonferroni correction) confirming statistical significance. The mean and SD of vessel length at 30–50 days of drug administration was 4.69 × 10^2^ ± 0.535 × 10^5^ mm.

Figure 6b shows the number of tumour cells at the end of the simulation for each condition. The lowest number of tumour cells was observed when the drug was administered for 30–50 days, with a mean number of tumour cells and SD of 0.471 × 10^5^ ± 0.0270 × 10^5^. On the other hand, the highest number of tumour cells was observed when the drug was administered for 30–60 days, with a mean number of tumour cells and SD of 0.724 × 10^5^ ± 0.207 × 10^5^. Although administration for 30–50 days resulted in 1.53 times as many tumour cells as those observed after administration for 30–60 days, *P* = 0.168 (Student's *t*-test, Bonferroni correction), the result was not statistically significant. Figure 6c,d shows a temporal change in the total length of newly sprouted vessels when the drug was administered for 30–40 and 30–60 days.

The final length of new vessels was found to decrease with increasing administration intervals. This may be due to the drug acting during the 50–60-day period, during which vascularization increased rapidly, for similar reasons as drug administration timing. Confirming the final number of tumour cells in each condition, the results indicated that they were 6.18% lower when the drug was administered for 20, rather than 10, days. This indicates that bevacizumab is more effective at inhibiting tumour growth when administered over a long period rather than an intensive short period. However, the final number of tumour cells was 53.5% higher when the drug was administered for 30 rather than 20 days.

According to the overall trend, Fig. 6c,d indicates increased vessel length was effectively suppressed when bevacizumab was administered for 30–60 days but new vessel length on day 40 was 17 mm longer than after drug administration for 30–60 days. In other words, more O_2_ was supplied to the tumour during the 30–40-day period, when the drug was administered for 30–60 days, than when the drug was administered for 30–60 days, indicating that inhibition effects on vasculature growth were insufficient for tumour growth inhibition.

According to this simulation, we propose that medication should be administered at a pace of no less than every other day to inhibit tumour growth. Finally, a combined comparison of all three conditions suggested that bevacizumab could most effectively inhibit tumour growth when administered with compatible duration and interval of administration.

## Conclusions and limitations

Some study limitations must be acknowledged. First, parameters in this simulation were adopted from several studies, rather than one, which may affect the results of this study. Second, vessels and tumours were fixed to each grid point and could not change their shape in response to the surrounding environment. Third, as this study focused predominately on sprouting vessel status changes, we did not consider tumour invasion or metastasis. Hence, we implemented only VEGF as one of the TAFs that promote angiogenesis while other molecules also function. Additionally, our results must be evaluated and validated in future clinical experiments and, if necessary, our model may be easily modified and updated.

This study developed a three-dimensional simulation model of angiogenesis and tumour growth. Specifically, we implemented the angiopoietin concentration balance, resulting in the reproduction of angiogenesis recession during vascular network formation. Furthermore, the efficacy of bevacizumab administration was evaluated using this model. Earlier administration showed higher tumour growth inhibition efficacy, with efficacy dependent on treatment interval even when the same dose was used. Following rigorous validation in the future, these results will contribute to the design of angiogenesis treatment protocols.

## Supplementary Information

Supplementary Information.

Supplementary Video S1.

Supplementary Video S2.

## Data Availability

Source code and the data used for the simulation are available upon request.
