# Supplementary material for: Mathematical simulation of tumour angiogenesis: angiopoietin balance is a key factor in vessel growth and regression
Source: Sci Rep. 2021 Jan 11;11:419. doi: 10.1038/s41598-020-79824-8 (PMC7801613; doi:10.1038/s41598-020-79824-8)
Supplement: Supplementary file 1 — Supplementary Information. [file 41598_2020_79824_MOESM1_ESM.docx]

**Supplementary Information for**

**Mathematical simulation of tumour angiogenesis: angiopoietin balance is a key factor in vessel growth and regression**

Hayato Yanagisawa^1^, Masahiro Sugimoto*^23^, Tomoyuki Miyashita^1^

^1^Waseda University, Faculty of Science and Engineering, Waseda University 3-4-1 Okubo, Shinjuku-ku, Tokyo 169-8555, Japan

^2^Department of Gastrointestinal and Pediatric Surgery, Tokyo Medical University, 6-7-1, Nishijinjuku, Shinjuku, Tokyo, 160-0023, Japan

^3^Institute for Advanced Biosciences, Keio University, Tsuruoka, Yamagata 997-0811, Japan

***To whom correspondence should be addressed:** Masahiro Sugimoto, PhD.

Research and Development Centre for Minimally Invasive Therapies, Medical Research Institute, Tokyo Medical University, 6-1-1, Shinjuku, Tokyo, 160-0022, Japan,

Tel: +81-235-29-0528, Fax: +81-235-29-0574

Email: mshrsgmt@tokyo-med.ac.jp

**Running title:** Elucidation of angiopoietin during angiogenesis

**Keywords:** angiogenesis, angiopoietin, bevacizumab, regression, simulation


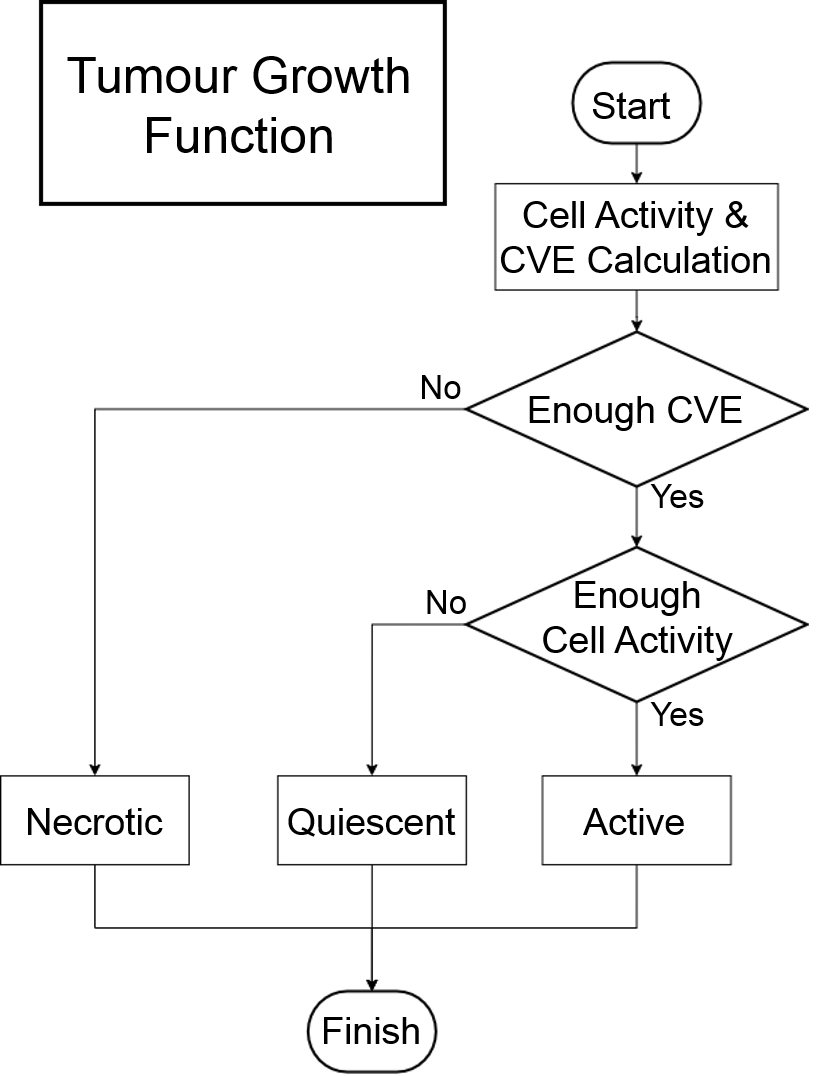


**Figure. S1 Flowchart of tumour growth.** A flowchart of tumour growth function details established during the simulation. Cell activity as calculated from the concentrations of oxygen and carbon dioxide. The amount of change in Cell Vital Energy (CVE), the energy stored in the cell for proliferation, depends on the cell activity of each tumour cell.


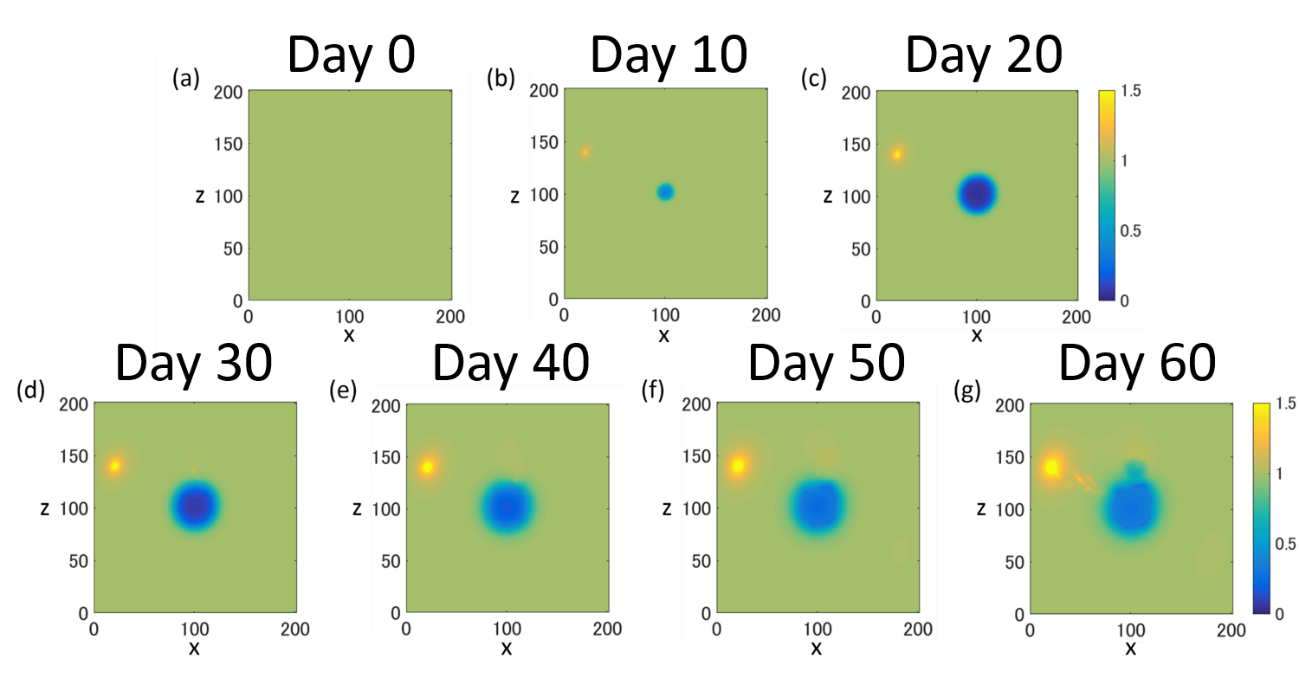


**Figure. S2 Temporal change of oxygen concentration distribution on days 0-60 (y = 100).** Snapshots of oxygen concentration at y = 100 on days 0 to 60. Oxygen was uniformly present throughout the initial phase. Oxygen was secreted by blood vessels and consumed by tumour cells.


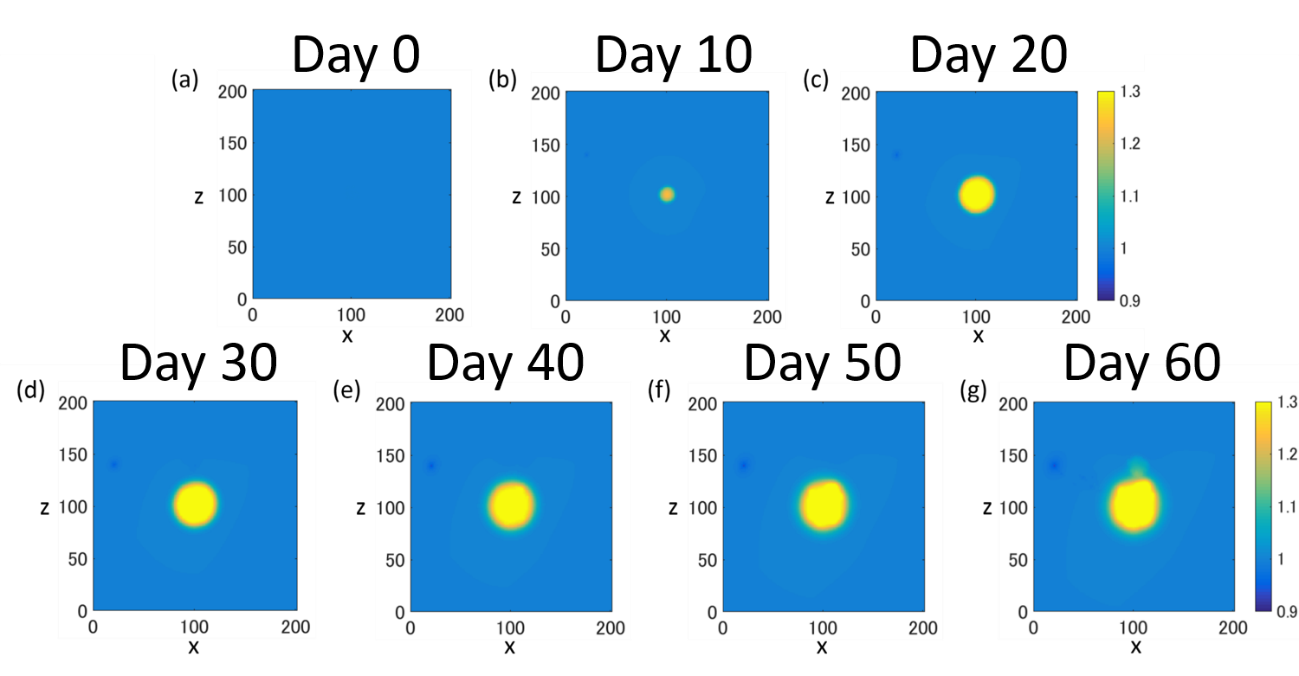


**Figure. S3 Temporal change of carbon dioxide concentration distribution on days 0-60 (y = 100).** Snapshots of carbon dioxide concentration at y = 100 on days 0 to 60. Carbon dioxide was secreted by tumour cells in the hypoxic state. It was not present in the initial phase.


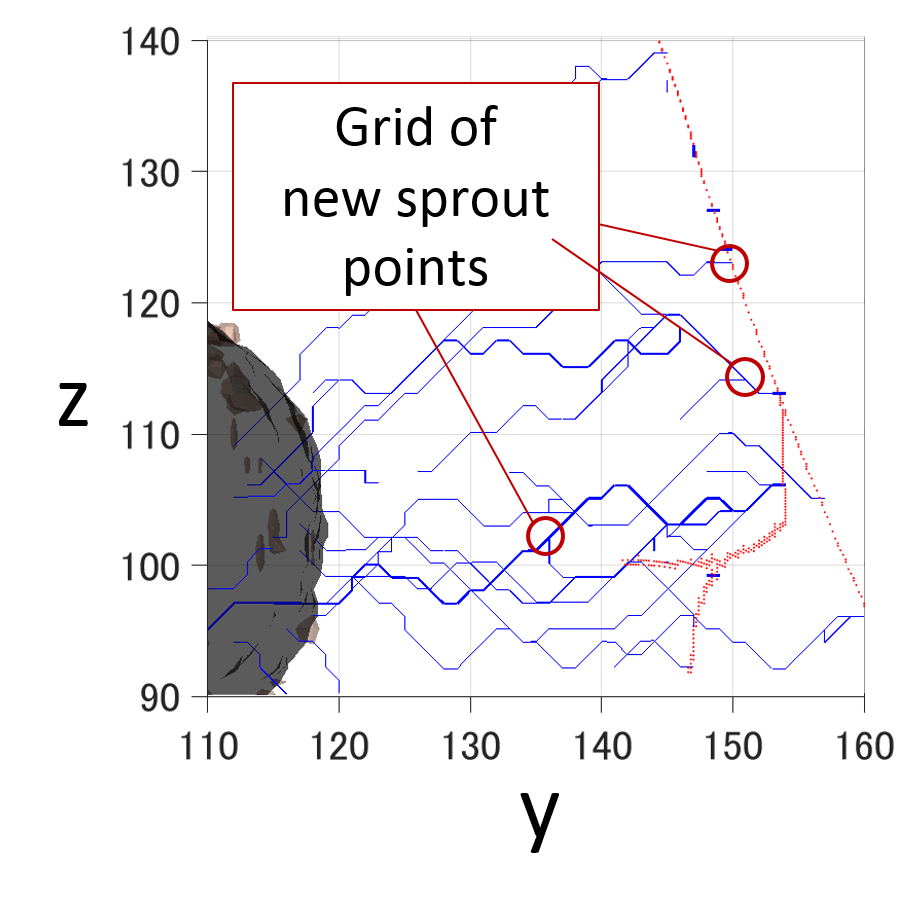


**Figure. S4 New vessel sprouting points on day 30 (x = 80-120).** This shows the sprouting points of new blood vessels. Red and blue curves show existing blood vessels and newly sprouted vessels, respectively.


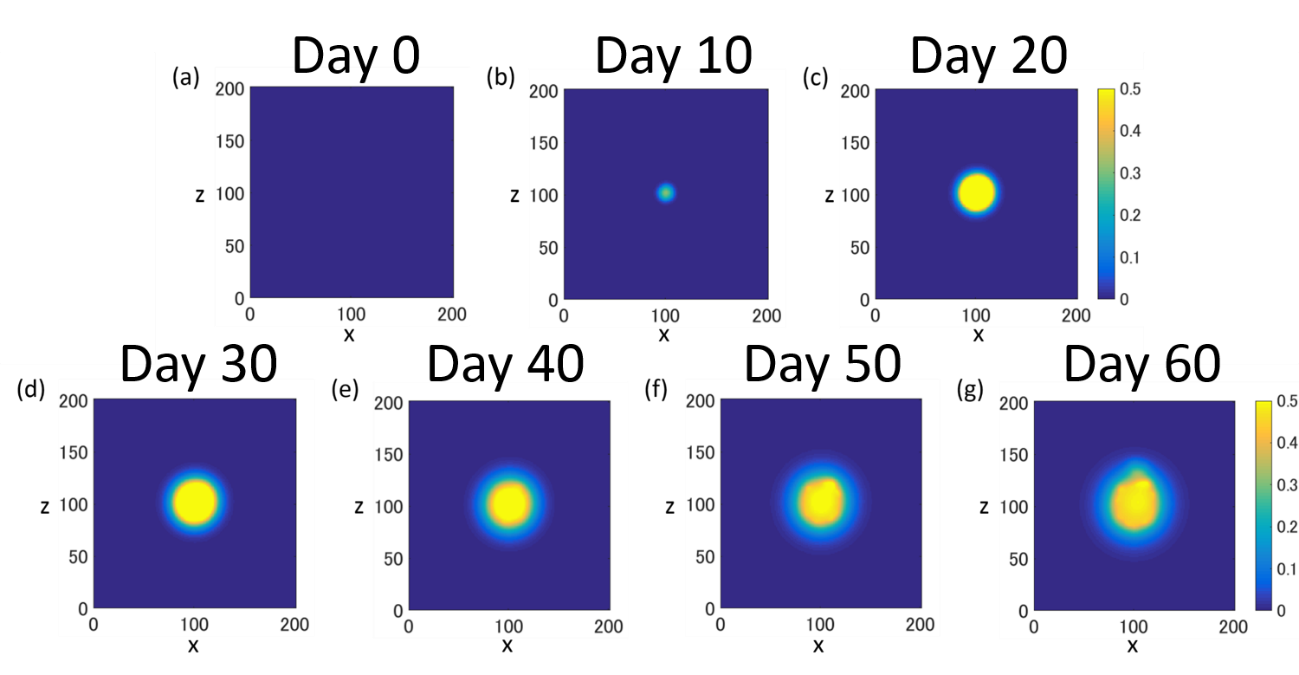


**Figure. S5 Temporal change of VEGF concentration distribution on days 0-60 (y = 100).** Snapshots of vascular endothelial growth factor (VEGF) concentration at y = 100 on days 0 to 60. VEGF was secreted by tumour cells in the hypoxic state. It was not present in the initial phase.

**Table. S1 Parameters used in angiogenesis simulations**

| Symbol | Value | Unit | Reference |
| --- | --- | --- | --- |
| $\lambda_{a_{2}\_0}$ | $4.6\times{10}^{-5}$ | $ml/({cm}^{3}s)$ | Estimated |
| $k_{BH\_0}$ | $3$.0 | $-$ | Estimated |
| $\alpha_{BH}$ | $1.2$ | $-$ | Estimated |
| $k_{rg\_0}$ | $1.8\times{10}^{-2}$ | $-$ | Estimated |
| $\alpha_{rg}$ | $1.3$ | $-$ | Estimated |
| $f$ | $2.5$ | $-$ | Estimated |
| $g$ | $2.5$ | $-$ | Estimated |
| $k_{active}$ | $1.0$ | $-$ | ^1^ |
| $k_{quiescent}$ | $0.1$0 | $-$ | ^1^ |
| $k_{AR1}$ | $1.0$ | $-$ | ^1^ |
| $k_{AR2}$ | $5.0\times{10}^{2}$ | $-$ | ^1^ |
| $k$ | $4.5\times{10}^{-15}$ | ${cm}^{2}/mmHg-sec$ | ^1^ |
| $p_{V}$ | $30$ | $mmHg$ | ^1^ |
| $p_{0}$ | $60$ | $mmHg$ | ^1^ |
| $n_{0}$ | $8.4$ | $mol/m^{3}$ |  |
| $w_{0}$ | $11$ | $mol/m^{3}$ | ^1^ |
| $c_{0}$ | $4.3\times{10}^{-4}$ | $kg/m^{3}$ | ^1^ |
| $a_{0}$ | $1.0\times{10}^{-3}$ | $mol/m^{3}$ | ^2^ |
| $e_{0}$ | $3.3\times{10}^{-8}$ | $m^{2}/s$ | ^2^ |
| h | 8.0 | $-$ | ^3^ |
| t_CVE_ | 30 | $-$ | ^1^ |

| Symbol | Value | Unit | Reference |
| --- | --- | --- | --- |
| $D_{n}$ | $8.0\times{10}^{-14}$ | $m^{2}/s$ | ^1^ |
| $\rho_{n0}$ | $6.8\times{10}^{-4}$ | $mol/(m^{3}s)$ | ^1^ |
| $\lambda_{n0}$ | $3.0\times{10}^{-5}$ | $ml/({cm}^{3}s)$ | ^1^ |
| $D_{w}$ | $4.0\times{10}^{-14}$ | $m^{2}/s$ | ^1^ |
| $\rho_{w0}$ | $1.0\times{10}^{-5}$ | $mol/(m^{3}s)$ | ^1^ |
| $\lambda_{w0}$ | $2.5\times{10}^{-5}$ | $ml/({cm}^{3}s)$ | ^1^ |
| $D_{c}$ | $1.2\times{10}^{-13}$ | $m^{2}/s$ | ^1^ |
| $\rho_{c0}$ | $2.0\times{10}^{-9}$ | $mol/(m^{3}s)$ | ^1^ |
| $\lambda_{c0}$ | $1.0\times{10}^{-13}$ | $ml/({cm}^{3}s)$ | Estimated |
| $D_{a_{1}}$ | $4.6\times{10}^{-16}$ | $m^{2}/s$ | ^2^ |
| $\rho_{a_{1}\_0}$ | $3.5{\times10}^{-1}$ | $mol/(m^{3}s)$ | Estimated |
| $\lambda_{a_{1}\_0}$ | $1.2\times{10}^{-6}$ | $ml/({cm}^{3}s)$ | Estimated |
| $D_{a_{2}}$ | $4.6\times{10}^{-17}$ | $m^{2}/s$ | ^2^ |
| $\rho_{a_{2}\_0}$ | $14$ | $mol/(m^{3}s)$ | Estimated |

**References**

[1] Tang, L. *et al.* Computational modeling of 3D tumor growth and angiogenesis for chemotherapy evaluation. *PLoS One.* **9**, e83962, 10.1371/journal.pone.0083962 (2014).

[2] Jackson, T. & Zheng, X. A cell-based model of endothelial cell migration, proliferation and maturation during corneal angiogenesis. *Bull. Math. Biol.* **72**, 830-868, 10.1007/s11538-009-9471-1 (2010).

[3] Plank, M. J., Sleeman, B. D. & Jones, P. F. A mathematical model of tumour angiogenesis, regulated by vascular endothelial growth factor and the angiopoietins. *J. Theor. Biol.* **229**, 435-454, 10.1016/j.jtbi.2004.04.012 (2004).

**Supplementary Videos**

**Video. S1** This shows angiogenesis-induced vascular network formation and tumour growth in the absence of drug administration. Red and blue curves show existing blood vessels and newly sprouted vessels, respectively.

**Video. S2** This shows diffusion of tumour-secreted VEGF in the absence of drug administration.
